# Scavenging ROS to Alleviate Acute Liver Injury by ZnO‐NiO@COOH

**DOI:** 10.1002/advs.202103982

**Published:** 2022-02-09

**Authors:** Xuan Wu, Shiyu Liu, Huanhuan Zhu, Zili Ma, Xiaohu Dai, Weiwei Liu

**Affiliations:** ^1^ Central Laboratory and Department of Laboratory Medicine Shanghai Tenth People's Hospital School of Medicine Tongji University Shanghai 200070 China; ^2^ State Key Laboratory of Pollution Control and Resource Reuse School of Environmental Science and Engineering Tongji University Shanghai 200092 China; ^3^ Shanghai Institute of Pollution Control and Ecological Security Shanghai 200092 China; ^4^ Department of Laboratory Medicine Longhua Hospital Shanghai University of Traditional Chinese Medicine Shanghai 200032 China

**Keywords:** acute liver injury, clinical therapy, interdisciplinary application, reactive oxygen species adsorption, ZnO‐NiO particles

## Abstract

Currently, the incidence of acute liver injury (ALI) is increasing year by year, and infection with coronavirus disease 2019 (COVID‐19) can also induce ALI, but there are still no targeted therapeutic drugs. ZnO–NiO particles is mainly used to clean up reactive oxygen species (ROS) in industrial wastewater, and it is insoluble in water. Its excellent properties are discovered and improved by adding shuttle‐based bonds to make it more water‐soluble. ZnO‐NiO@COOH particles are synthetically applied to treat ALI. The *p‐n* junction in ZnO–NiO@COOH increases the surface area and active sites, thereby creating large numbers of oxygen vacancies, which can quickly adsorb ROS. The content in tissues and serum levels of L‐glutathione (GSH) and the GSH/oxidized GSH ratio are measured to assess the capacity of ZnO–NiO@COOH particles to absorb ROS. The ZnO–NiO@COOH particles significantly reduce the expression levels of inflammatory factors (i.e., *IL‐1*, *IL‐6*, and *TNF‐α*), macrophage infiltration, and granulocyte activation. ZnO‐NiO@COOH rapidly adsorb ROS in a short period of time to block the generation of inflammatory storms and gain time for the follow‐up treatment of ALI, which has important clinical significance.

## Graphical abstract

This article designs and improves ZnO–NiO@COOH particles, which generate oxygen vacancies to absorb reactive oxygen species (ROS). In vivo and in vitro experiments confirm that the spherical petal‐like ZnO‐Nio@COOH material can effectively treat acute liver injury. In conclusion, ZnO‐NiO@COOH alleviates liver injury by efficiently adsorbing ROS and inhibiting the production of inflammatory factors.

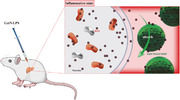

## 1 Introduction

Acute liver injury (ALI) is acute and severe cases are likely to cause death.^[^ ^1^, ^2^, ^3^ ^]^ It has been reported that infection with coronavirus disease 2019 induces severe ALI and lacks effective treatment.^[^ ^4^ ^]^ According to the World Health Organization, the incidence of ALI continues to increase due to drug abuse, the heavy use of pesticides, environmental pollution, and unhealthy lifestyle habits.^[^ ^1^, ^2^, ^3^ ^]^ There are three potential outcomes to ALI: disease resolution, progression to chronic liver injury, and progression to acute liver failure.^[^ ^2^, ^5^ ^]^ However, at present, there is a lack of effective methods to prevent and treat acute liver disease in clinical practice.^[^ ^6^, ^7^ ^]^

Significant amounts of reactive oxygen species (ROS) are produced during the onset and progression of ALI.^[^ ^8^, ^9^, ^10^ ^]^ In general, hepatocytes contain relatively higher numbers of mitochondria as compared to other cell types.^[^ ^10^, ^11^, ^12^ ^]^ As the power producers of the cell, mitochondria are not only the main site of ROS production, but also the most vulnerable organelles to oxidative damage.^[^ ^13^, ^14^, ^15^ ^]^ Due to the abundance of mitochondria in hepatocytes, the liver is the main target of ROS.^[^ ^15^ ^]^ In response to excessive ROS, hepatocytes produce large numbers of inflammatory factors, such as tumor necrosis factor *TNF‐α*, nuclear factor *NF‐κB*, and interleukin *IL‐1β*, in addition to apoptosis‐related proteins, such as caspase‐3 and ‐9, resulting in a vicious circle that exacerbates damage to liver cells.^[^ ^11^, ^16^, ^17^, ^18^ ^]^ ROS and apoptosis play key roles in ALI.^[^ ^17^, ^19^, ^20^] Although the application of antioxidants, such as N‐acetylcysteine, can provide effective treatment, there is a lack of liver‐specific therapeutic drugs.^[^ ^8^, ^21^, ^22^ ^]^ Early treatment within the first 8 h of the occurrence of ALI is essential for favorable outcomes.^[^ ^20^, ^22^, ^23^ ^]^ Therefore, new therapeutic methods and strategies are key to the treatment of drug‐induced ALI. The results of this study promoted a better understanding of the pathogenesis of ALI and provided a new treatment strategy.

Recent studies have reported outstanding advantages of metal oxide semiconductors (MOSs) for the treatment of ALI,^[^ ^24^, ^25^, ^26^ ^]^ especially the absorption of ROS. MOSs are generally classified as the *n‐*type or *p‐*type with the main carriers being electrons and holes, respectively.^[^ ^27^, ^28^ ^]^ Generally, *n‐*type MOSs are highly sensitive with fast response recovery, but the operating temperature is high and the selectivity is poor.^[^ ^29^ ^]^ In contrast, *p*‐type MOSs have a lower operating temperature and long‐term stability, but sensitivity is relatively lower. Therefore, many studies have focused on combining the *n‐* and *p*‐types to form a *p‐n* junction in order to obtain materials with the advantages of both. The formation of a *p‐n* structure can effectively improve the ROS adsorption performance of the material.^[^ ^30^ ^]^ The *p‐n* junction controls resistance by regulating the interface potential energy barrier and narrowing the conduction channel, thereby improving sensor performance.^[^ ^31^ ^]^ ZnO and NiO can be used as a combination of the *n‐* and *p‐*type components to form a representative *p‐n* junction material.^[^ ^32^, ^33^, ^34^ ^]^ The *p‐n* junction of the ZnO/NiO composite increases the surface area, active sites, and creates large numbers of oxygen vacancies, so as to achieve chemical adsorption and dissociation of ROS.^[^ ^35^ ^]^ In this study, a ZnO/NiO composite was manufactured for the treatment of drug‐induced ALI. Our material enters the body and does not directly enter the cell, which is more conducive to its metabolism and reduces the residue in the body.

ZnO–NiO@COOH can exert an anti‐inflammatory function by scavenging free radicals, thereby reducing necrosis of liver cells. In vitro and in vivo experiments together demonstrated the ability of ZnO–NiO@COOH particles to adsorb ROS efficiently. Animal experiments have shown that ZnO‐NiO@COOH is rapidly enriched to the liver after entering the body through blood circulation. Macrophages are activated to produce large amounts of inflammatory factors after antigen invasion.^[^ ^8^ ^]^ ZnO‐NiO@COOH material enriched in the liver can significantly increase the serum peroxidase level, while reducing the expression level of pro‐inflammatory factors, thus effectively alleviating the degree of liver congestion and necrosis. At present, treatment of ROS‐induced cellular damage remains difficult.^[^ ^36^, ^37^ ^]^ The results of the present study showed that the rapid absorption of large amounts of ROS by ZnO–NiO@COOH can effectively slow down the generation of inflammatory storms and reduce the release of inflammatory factors and achieve the best effect when the molar ratio of Zn to Ni is 0.2. The performance of ZnO_0.2_–NiO@COOH particles has been improved by increasing the surface area and active sites, thereby creating a large number of oxygen vacancies, which can quickly absorb large amounts of ROS for the treatment of ALI. The use of ZnO–NiO has achieved remarkable results in absorbing ROS in other fields, which have provided new ideas for the treatment of ALI. Hence, Our innovative synthesis of ZnO_0.2_–NiO@COOH particles. ZnO_0.2_‐NiO@COOH has the potential to be a new approach for a safe and efficient treatment for the prevention and treatment of ALI.

## 2 Results

### 2.1 Scanning Electron Microscopy (SEM), Energy Dispersive Spectroscopy (EDS), and X‐ray Diffractometry (XRD) Images of the ZnO_0.2_–NiO@COOH Particles

**Figure 1:**
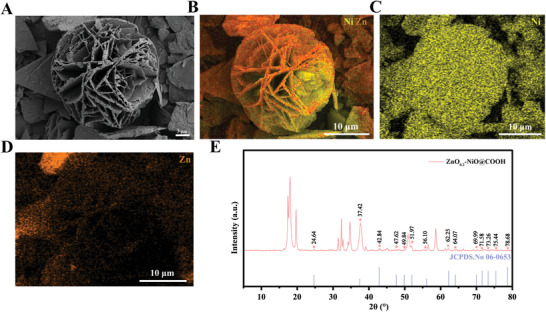
The morphology and composition of ZnO_0.2_–NiO@COOH. A) SEM images. B–D) Energy dispersive spectroscopy. E) XRD pattern.

As shown in **Figure** **1**A, ZnO_0.2_–NiO@COOH is a petal‐shaped porous pellet with an ultra‐thin porous spherical structure that provided a rich interface between the material and electrolyte. ZnO_0.2_–NiO@COOH fully mixed with blood, which was conducive to the adsorption of ROS from the blood to the surface of the particle. Ni and Zn were distributed on the spherical structure of ZnO_0.2_–NiO@COOH (Figure 1A–D). In addition, the content of Ni was much higher than that of Zn, which was also consistent with the molar ratio of Ni to Zn used in the production process. NiO and ZnO were not simply stacked, but rather a composite of Ni and Zn, which was consistent with JCPDS.No 06–0653 (Figure 1E). Therefore, *p‐n* junctions were formed at the interface of Ni and Zn, which was conducive to the adsorption of ROS.

### 2.2 X‐ray Photon Spectroscopy (XPS) Spectrum of the ZnO_0.2_–NiO@COOH Particles

**Figure 2:**
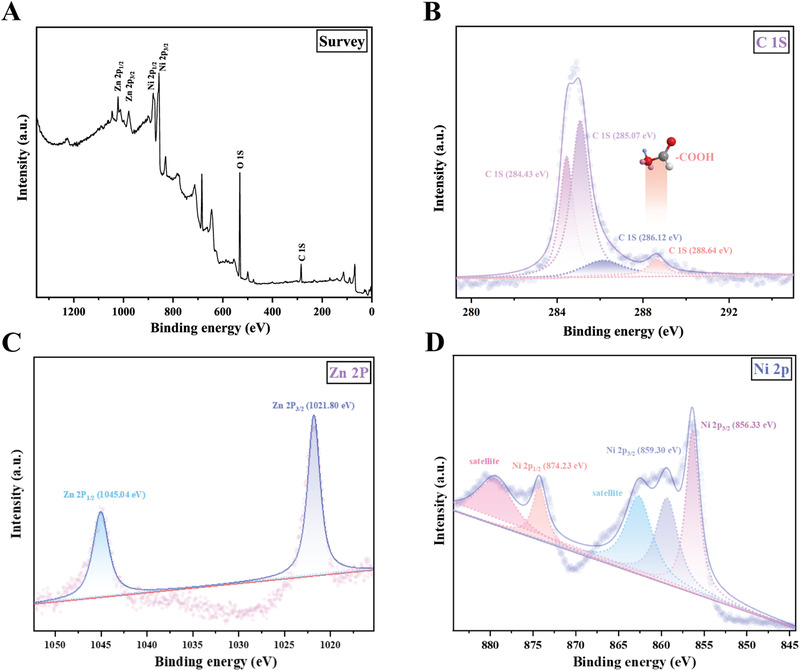
XPS spectra of ZnO_0.2_–NiO@COOH. A) XPS full spectrum. B,C) 1*s* spectra. (C) Zn 2*p* spectra. D) Ni 2*P* spectra.

The full scan spectrum presented in **Figure** **2**A shows that the ZnO_0.2_–NiO@COOH particle was composed of Zn, Ni, O, and C, which was consistent with the EDS results. The binding energy of the C 1*s* peak at 284.5 eV was used as a reference for calibration (Figure 2B). Notably, a new C 1*s* peak appeared at 288.6 eV, which was a carboxyl group, indicating that a carboxyl group was successfully grafted onto the ZnO_0.2_–NiO@COOH particle. A high‐resolution XPS spectrum of Zn 2*p* is shown in Figure 2C. The spin‐orbit split of Zn 2*p* was 23.2 eV, which was consistent with the corresponding value of pure ZnO, indicating the presence of Zn^2+^. As shown by the high‐resolution *XPS* spectrum of Ni 2*p* presented in Figure 2D, Ni 2*p*3/2 was located at 856.3 and 859.3 eV, indicating the presence of Ni*^3+^*, while Ni 2*p*1/2 was located at 874.2 eV, indicating the presence of Ni*^2+^*. As compared to pure NiO, the binding energy of Ni 2*p* showed a significant shift to the left, likely because NiO was a *p‐*type oxide and ZnO was an *n‐*type oxide. When NiO was combined with ZnO, due to the difference in the Fermi level, Ni^2^ *^+^* absorbed electrons from Zn^2^ *^+^*. The generation of *p‐n* junctions caused oxygen vacancies. The combination of *p‐n* junctions and multivalent transitional metal ions promoted the adsorption of ROS.

### 2.3 Fourier Transform Infrared (FTIR) Spectrum and Hydrophilicity of ZnO_0.2_–NiO@COOH

**Figure 3:**
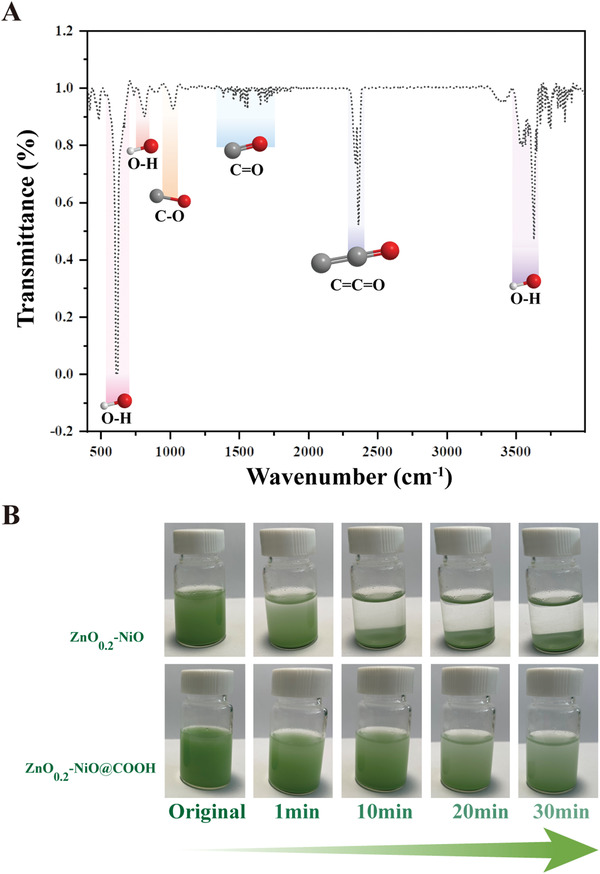
FTIR spectrum and hydrophilicity analysis. A) FTIR spectrum of ZnO_0.2_–NiO@COOH. B) Settlement diagram of ZnO_0.2_–NiO and ZnO_0.2_–NiO@COOH over time.

As shown in **Figure** **3**A, stretching and vibration of O—H occurred at 615, 1018, and 3628 cm^−1^. The results of hydrophilicity analysis of the ZnO_0.2_–NiO@COOH particles are presented in Figure 3B. The sedimentation performance was analyzed by adding 15 mg of ZN and ZnO_0.2_–NiO@COOH to 25 mL of deionized water, then shaking to uniformity, and allowing to stand. As shown in Figure 3B, ZN precipitated rapidly at 1 min and almost all had precipitated by 10 min. In contrast, the sedimentation of ZnO_0.2_–NiO@COOH grafted with carboxyl groups began to slowly settle at 1 min and was still incomplete after 30 min, indicating that the hydrophilic performance was greatly improved. This structure facilitated ZnO_0.2_–NiO@COOH to enter the lesion via the blood circulation. Then, after being absorbed in the lesion, ROS was excreted from the body and did not accumulate.

### 2.4 The ROS Adsorption Capacity of ZnO–NiO@COOH

**Figure 4:**
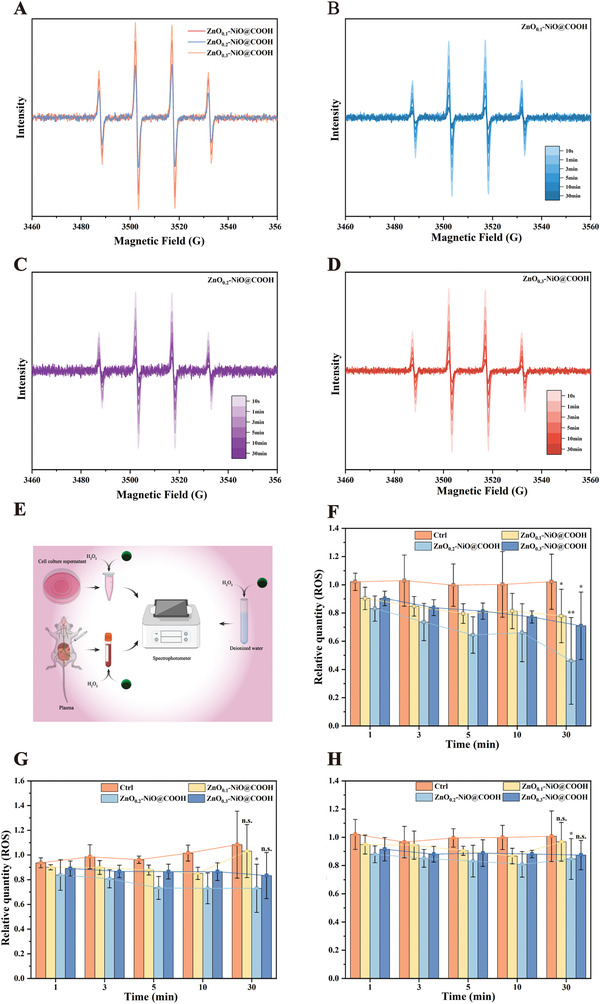
EPR tests of OH·‐radicals detection. A) The EPR of the three materials after adsorbing OH*·‐* in deionized water for 30 min. B) EPR of OH*·‐* adsorption of ZnO_0.1_‐NiO@COOH in deionized water with time. C) EPR of OH*·*‐ adsorption of ZnO_0.2_‐NiO@COOH in deionized water with time. D) EPR of OH·‐ adsorption of ZnO_0.3_‐NiO@COOH in deionized water with time. E) Schematic diagram of three different operating conditions. F–H) Detect the ability of particles to absorb ROS in deionized water, cell culture supernatant and plasma (*n =* 3, *p < 0.05, **p < 0.01, n.s.>0.05).

In order to further verify the in vitro ROS adsorption capacity of ZnO–NiO@COOH particles, we performed electron paramagnetic resonance (EPR) assays and in vitro adsorption experiments. As shown in **Figure** **4**A, the three materials are put into a fixed concentration (100 um) of H_2_O_2_, and after 30 min of adsorption, when the molar ratio of Zn to Ni is 0.2, the strength of EPR is the lowest. It shows that the adsorption capacity of ZnO_0.2_‐NiO@COOH is the best. It can be seen from Figure 4B–D that all three materials can gradually adsorb ROS. However, the absorption intensity of ZnO_0.2_‐NiO@COOH changes the most in the first 1 min. It shows that ZnO_0.2_‐NiO@COOH has the best ability to absorb ROS in a short time. Figure 4E is a schematic diagram of three different conditions. Figure 4F–H is the changes in the adsorption of ROS in deionized water, cell culture supernatant, and plasma. The results show that all materials have adsorption capacity and the adsorption effect of ZnO_0.2_‐NiO@COOH is the best, which is consistent with the EPR results. However, the adsorption effect of the material in deionized water is better than that in cell culture supernatant and plasma. The ROS absorption capacity of the three particles was verified at the in vitro cell level. The ROS in the cell can oxidize the non‐fluorescent Dichlorodihydrofluorescein‐diacetate fluorescent probe to generate fluorescent Dichlorofluorescein (DCF). By detecting the fluorescence intensity of DCF, the level of ROS in the cell can be known. As the results show, the ROS absorption capacity of ZnO_0.2_‐NiO@COOH is the best (Figure S1A,B, Supporting Information).

### 2.5 Biosafety of ZnO_0.2_–NiO@COOH

**Figure 5:**
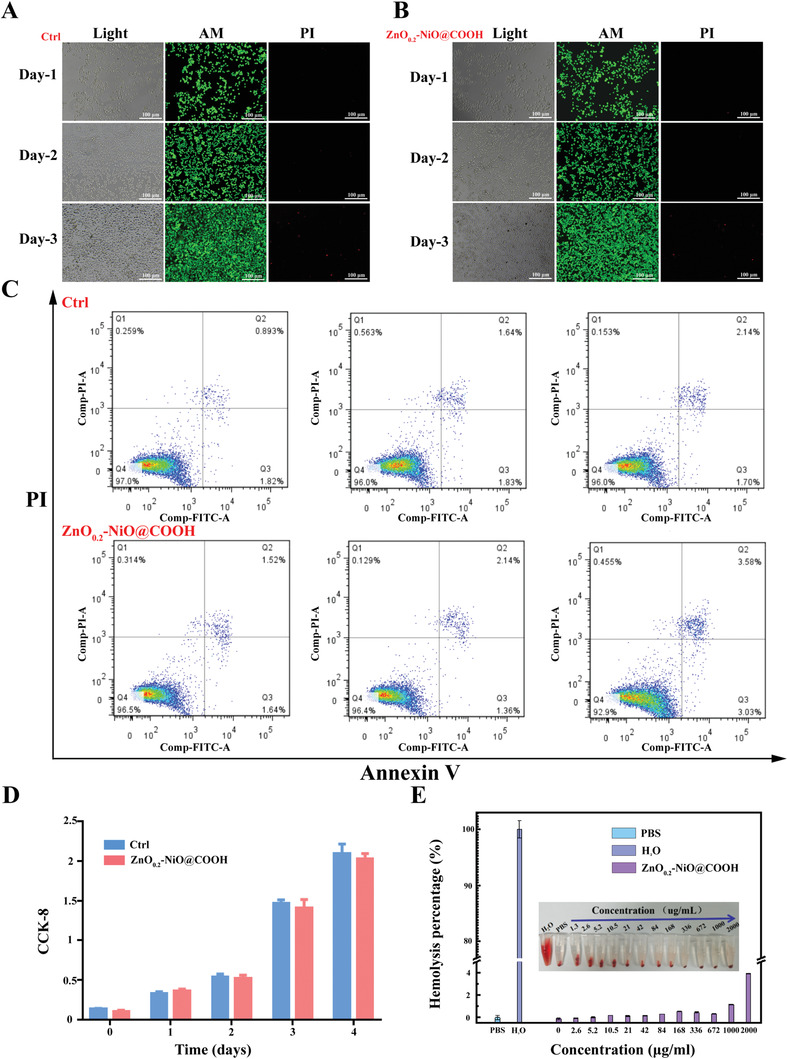
Biosafety of ZnO_0.2_‐NiO@COOH. A,B) observation of the morphology and growth status of PLC cells under normal conditions. Calcein‐AM/PI‐stained live and dead cells (calcein excitation and emission wavelengths: 490 and 515 nm, respectively; PI excitation wavelength: 488 and 545 nm, respectively, emission wavelength: 617 nm). C) At 24, 48, and 72 h, AnnexinV‐FITC flow cytometry was used to detect early apoptotic cells (Q3), late apoptotic cells (Q2), necrotic cells (Q1), and live cells (Q4). D) *CCK‐8* detection of cell proliferation on days 0, 1, 2, 3, and 4 (*n* = 3, *p* > 0,05). E) Hemolysis testing results.

Prior to clinical application, the safety and cytotoxicity of biomaterials must be assessed. To evaluate potential cytotoxicity, the growth, proliferation, and apoptosis of hepatoma cell lines were monitored after the addition of ZnO_0.2_–NiO@COOH to the culture medium (**Figure** **5**A–C). Combined use of Annexin V and PI distinguished live cells from early apoptotic cells, late apoptotic cells, and dead cells (Figure 5C). The primary liver cancer (PLCs) were cultured with ZnO_0.2_–NiO@COOH for 96*h* and cell growth was recorded every 24 h. As compared with the control group, ZnO_0.2_–NiO@COOH had no effect on cell proliferation and viability (Figure 5D). Hemolysis experiments conducted with different concentrations of ZnO_0.2_–NiO@COOH confirmed that it did not cause hemolysis at higher concentrations, which further confirmed the safety of the particles (Figure 5E). Taken together, these results confirmed the low cytotoxicity of ZnO_0.2_–NiO@COOH, which is of great significance for further animal experiments.

Upon entry to the human body, foreign materials can destroy normal defense mechanisms. Materials with high surface activity can easily pass through the blood‐brain barrier and skin to reach various organs through the blood circulation. Therefore, it is vital to assess the safety of new biomaterials.

Animal experiments were conducted in order to further verify the biological safety of the ZnO_0.2_–NiO@COOH. ZnO_0.2_–NiO@COOH were injected into mice and liver samples were collected at 0, 24, 48, and 72 h and then stained with hematoxylin and eosin (HE) staining to assess the effects of it after different times of exposure (Figure S2A, Supporting Information). Serum levels of alanine transaminase (ALT) and aspartate transaminase (AST) were measured to test for liver damage. The results showed that there was no significant difference in the liver function of mice at different time points, thereby confirming that ZnO_0.2_–NiO@COOH caused no liver damage (Figure S2B, Supporting Information). The expression levels of various inflammatory factors (i.e., *IL‐1β, IL‐6*, and *TNF‐α*) in liver tissue were measured and the results showed that ZnO_0.2_–NiO@COOH did not cause inflammation (Figure S2C, Supporting Information). These results confirmed that ZnO_0.2_–NiO@COOH was biologically safe and did not cause liver congestion, necrosis, or inflammation.

### 2.6 The Protective Effect of ZnO_0.2_–NiO@COOH Against ALI

**Figure 6:**
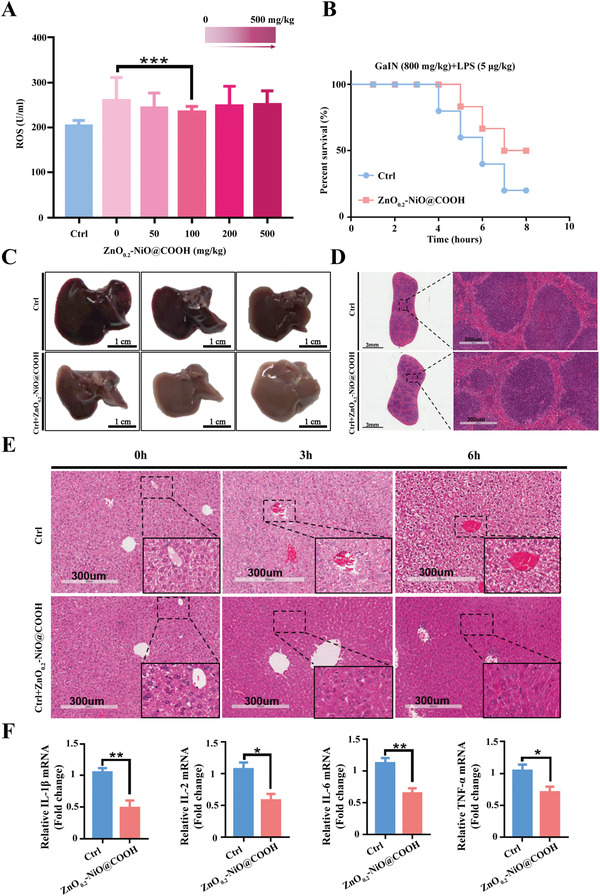
ZnO_0.2_–NiO@COOH effectively relieves ALI. A) ROS absorption levels at different concentrations of ZnO_0.2_–NiO@COOH during ALI (*n =* 5, ***p < 0.001). B) Statistical analysis of survival time (*n =* 5, *p < 0.05). C) Schematic diagram of the liver (*n = 5*). D,E) HE staining to observe congestion and necrosis (*n =* 5). F) Expression levels of inflammatory factors in liver tissue (*n* = 3, *p < 0.05, **p < 0.01).

Animal experiments were conducted in order to verify the protective effect of ZnO–NiO@COOH against drug‐induced ALI. Animals model of ALI was constructed by intraperitoneal injection of galactosamine (800 mg kg^−1^ body weight) and lipopolysaccharide (5 µg kg^−1^ body weight). Before the formal experiment, we constructed ALI model to give six different ZnO–NiO@COOH concentrations, and the optimal dosing was determined by detecting the ROS levels in serum. According to the results of **Figure** **6**A, it is confirmed that the concentration of 100 mg kg^−1^ is the best for the treatment of ALI. We treated ALI animals with three ratios of ZnO‐NiO@COOH (ZnO_0.1_–NiO@COOH, ZnO_0.2_–NiO@COOH, and ZnO_0.3_–NiO@COOH) and the results were consistent with in vitro experiments. ZnO_0.2_–NiO@COOH has the best protective effect on ALI (Figure S3A–E, Supporting Information). ZnO_0.2_–NiO@COOH (100 mg kg^−1^) was injected into the tail vein of the experimental mice, while the control mice received the same volume of sodium chloride solution. As shown in Figure 6B, the overall survival time of mice in the ZnO_0.2_–NiO@COOH group was prolonged, while the mortality rate was reduced (*p < *0.05). Further analyses of mouse liver and spleen samples showed that liver congestion and necrosis were significantly reduced in the ZnO_0.2_–NiO@COOH group as compared with the control group (Figure 6C). HE staining of spleen samples to observe bleeding and necrosis revealed lower inflammatory cell infiltration in the treatment group (Figure 6D). Furthermore, HE staining of liver samples collected from the two groups of mice at 0, 3, and 6 h showed reduced hemorrhaging and necrosis in the liver of the ZnO_0.2_–NiO@COOH group along with lower congestion of the liver sinusoids (Figure 6E). Meanwhile, the expression levels of the inflammatory factors *IL‐1β, IL‐2, IL‐6*, and *TNF‐α* in the liver tissues of mice in the ZnO_0.2_–NiO@COOH group were significantly decreased (Figure 6F). Overall, these results confirmed that ZnO_0.2_–NiO@COOH effectively alleviated drug‐induced ALI, although the underlying mechanism remains unclear.

Lipopolysaccharides are components of the cell wall surface of Gram‐negative bacteria that can stimulate immune cells, including macrophages, to release inflammatory factors, which cause apoptosis and necrosis of hepatocytes. Galactosamine‐induced secondary liver injury results in the activation of macrophages and granulocytes, and the inflammatory response causes apoptosis of hepatocytes. Free radicals play an important role in galactosamine‐mediated necrosis. In order to further explore the mechanism of ZnO_0.2_–NiO@COOH in the alleviation of ALI, in‐depth experimental studies were conducted.

**Figure 7:**
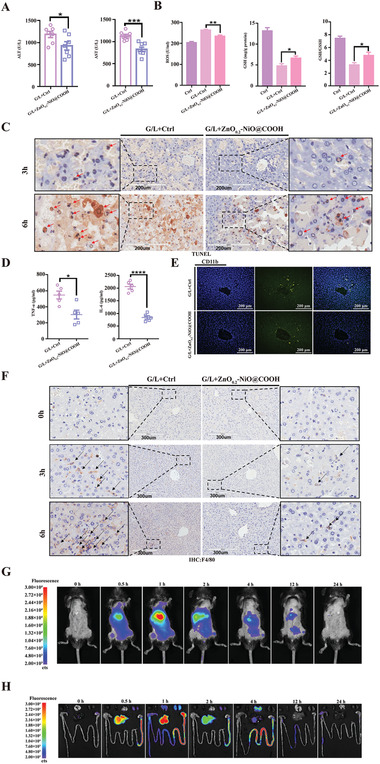
ZnO_0.2_–NiO@COOH relieves ALI by reducing the production of inflammatory factors. A) ALT and AST detection to assess liver function (*n* = 8, *p < 0.05, ***p < 0.001). B) Detection of serum ROS, GSH content, and the GSH/GSSH ratio (*n* = 5–8, *p <> 0.05, **p < 0.01). C) TUNEL detection of apoptotic cells (*n* = 3). D) Detection of serum inflammatory factor levels (*n* = 5, *p < 0.05, ****p < 0.0001). E) Immunofluorescence staining (*n* = 3). F) Immunohistochemical staining (n = 3). G) Animal in vivo imaging. H) Distribution of ZnO_0.2_‐NiO@COOH in organs (*n* = 3/group).

Serum biochemical testing showed that as compared with the control group, the increases in serum levels of *ALT* and *AST* in the ZnO_0.2_–NiO@COOH group were relieved (**Figure** **7**A). In addition, testing of serum levels of ROS, glutathione reductase (GSH), oxidized glutathione (GSSH), and the GSH*/*GSSH ratio showed that ZnO_0.2_–NiO@COOH effectively absorbed ROS produced by ALI in mice (Figure 7B). TUNEL staining revealed that the number of apoptotic cells in the ZnO_0.2_–NiO@COOH group was significantly reduced (Figure 7C). Blood analysis further confirmed that ZnO_0.2_–NiO@COOH effectively reduced the expression and secretion of inflammatory factors (e.g., *TNF‐α* and *IL‐6*) (Figure 7D). Immunohistochemical staining of *CD11b*, which is expressed on the surfaces of macrophages, granulocytes, and dendritic cells, showed that ZnO_0.2_–NiO@COOH reduced the activation of immune cells (Figure 7E). *F*4/80, the murine homolog of mucin‐like hormone receptor 1, is a highly glycosylated G protein‐coupled receptor commonly used as a surface marker of mouse macrophages. Immunohistochemical staining to detect the infiltration and expression of macrophages showed that ZnO_0.2_–NiO@COOH effectively reduced the infiltration of macrophages (Figure 7F). Collectively, these experimental results confirmed that ZnO_0.2_–NiO@COOH effectively adsorbed ROS and alleviated the damage caused by ALI by inhibiting the activation of immune cells and the infiltration of macrophages, reducing the secretion of inflammatory factors.

Finally, we wanted to further understand the distribution and enrichment of ZnO‐NiO@COOH in vivo. We combined ZnO‐NiO@COOH with the luciferase dye Indocyanine green (ICG) to demonstrate the distribution trajectories by in vivo imaging in animals. The results of in vivo imaging showed that material was enriched in the liver immediately after entering the animal body and reached the maximum peak at about 1 h (Figure 7G). The accumulation of ZnO‐NiO@COOH in the liver gradually decreased after 1 h, and it was completely metabolized out of the body after 24 h. Furthermore, we also examined the distribution and enrichment of ZnO‐NiO@COOH in the heart, liver, spleen, lung, kidney, stomach, and colorectum. As shown in Figure 7H, ZnO‐NiO@COOH is the first to be enriched in the liver after entering the body. Through circulatory metabolism, it is gradually eliminated from the body through the digestive tract and does not remain in the body.

## 3 Discussion and Conclusion

In summary, ZnO_0.2_‐NiO@COOH can efficiently absorb ROS and block the generation of inflammatory storms to save time for ALI treatment, which has important clinical application value.

Interdisciplinary and cross‐application is the inevitable trend of future scientific development.^[^ ^38^ ^]^ Precision medicine is the most popular concept in recent years. From diagnosis, treatment to prognosis, all aspects of medical treatment are looking for suitable ways to achieve precision medicine. Now, medicine with nanomaterials and particles as the carrier is becoming a new favorite in the pharmaceutical field, affecting the original drug research and development model. If the nanomaterials or particles themselves can exert curative effects, it can shorten the development time and improve the treatment efficiency. Therefore, The ZnO_0.2_–NiO@COOH have important clinical application value for the treatment of ALI. Moreover, further studies are needed to assess the effects of ZnO_0.2_–NiO@COOH against other acute ROS injury‐related diseases, such as liver ischemia‐reperfusion injury, hypovolemic shock, acute poisoning, and liver failure, as well as chronic ROS injury diseases, such as cancer, atherosclerosis, and diabetes.^[^ ^36^, ^39^, ^40^, ^41^, ^42^, ^43^, ^44^ ^]^ GSH is the main direct reducing agent in the cell and catalyzes reduction reactions and is also essential for glutathione peroxidase (GPx) catalysis of peroxide reduction.^[^ ^45^, ^46^ ^]^ The content of GSH in the cell is about tenfold greater than that of GSSH.^[^ ^45^ ^]^ The results of the present study confirmed that ZnO_0.2_–NiO@COOH quickly and effectively absorbed ROS in the body, while increasing the content of GSH and the GSH*/*GSSH ratio. ZnO_0.2_–NiO@COOH themselves are not biologically toxic, and will not cause damage to animals and cells to produce ROS. In addition, ZnO_0.2_–NiO@COOH significantly decreased the expression levels of *IL‐1, IL‐6*, and *TNF‐α* in liver tissues. The adsorption of ROS by ZnO_0.2_–NiO@COOH not only had anti‐oxidation effects, but also inhibited the activation of immune cells and reduced the expression of apoptosis‐related proteins.

Oxidative stress is closely linked to inflammation.^[^ ^47^, ^48^ ^]^ Studies have reported that high concentrations of ROS activate Toll‐like receptors (TLRs), which play regulatory roles in the adaptive immune response.^[^ ^49^, ^50^, ^51^ ^]^ Hepatocytes express functional *TLR4*, while activated macrophages produce more *IL‐1β*, *IL‐6*, *TNF‐α*, and through the *TLR4* pathway. It has been reported that oxidative stress during shock activated native tissue macrophages via the recruitment and accumulation of *TLR4* on the cell surface.^[^ ^50^, ^51^ ^]^ Activated *TLR4* can also up‐regulate other *TLRs* to form positive feedback loops, which further aggravate cellular damage.^[^ ^50^ ^]^ In addition, ROS can activate the nuclear factor erythroid 2‐related factor 2 (Nrf2)/antioxidant responsive element signaling pathway, in which mitogen‐activated protein kinases, protein kinase C, and phosphatidyl alcohol‐3‐kinase participate in several protein kinase pathways.^[^ ^52^, ^53^ ^]^ In‐depth mechanism exploration still needs more experiments to verify. Although the results of this study partially revealed the mechanisms underlying the ability of ZnO_0.2_–NiO@COOH to absorb ROS, the specific pathways remain unclear, thus further studies are warranted.

All in all, ZnO_0.2_–NiO@COOH particles can effectively adsorb ROS in a short time and relieve ALI, which has important clinical significance for clinicians to treat acute liver damage.

## 4 Experimental Section

### Preparation of ZnO–NiO

The ZnO_0.2_–NiO@COOH particles were produced by dissolving 12g of CO (NH_2_)_2_, 7.4 g of NH_4_F, 29.5 g of Ni (NO_3_)_2_, and 2.97 g of Zn (NO_3_)_2_ in 1 L of deionized water to obtain mixture A. The molar ratio of Ni to Zn was set to 5:1. Then, mixture A was added to a Teflon‐lined stainless steel hydration reactor and heated to 140 °C for 2 h. Afterward, the mixture was centrifuged to obtain a precipitate, which was vacuum dried at 60 °C to obtain Zn (OH)_2_‐Ni (OH)_2_. The dried Zn (OH)_2_‐Ni (OH)_2_ was heated to 500 °C at a rate of 5 °C min^−1^ for 2 h in a tube furnace with the use of nitrogen gas, which was introduced at a rate of 50 mL min^−1^. This process was followed by annealing the nitrogen for 4 h to obtain a composite of ZnO‐NiO.

### Preparation of ZnO–NiO@COOH

For the preparation of the final ZnO_0.2_–NiO@COOH composite, 2 g of ZnO‐NiO was added to 60 mL of a solution of ethanol and deionized water (1:1) along with 0.2 mL of 3‐mercaptopropionic acid. After magnetically stirring for 24 h, 0.5 g of N'N‐carbonyldiimidazole was added to the mixture, which was magnetically stirred for an additional 2 h and then centrifuged to obtain the ZnO‐NiO@COOH composite ZnO_0.2_–NiO@COOH. Similarly, to examine the effect of differences in Zn loading, the same procedure was repeated with varied Zn content (1.48, 2.97, and 4.46) so that the molar ratio of Zn to Ni was 0.1, 0.2, and 0.3 respectively. These samples were respectively denoted as ZnO_0.1_–NiO@COOH, ZnO_0.2_–NiO@COOH, and ZnO_0.3_–NiO@COOH.

### Material Characterization

The surface morphology of the ZnO‐NiO@COOH particles was analyzed via scanning electron microscopy (SEM) (S‐4800; Hitachi Corporation, Tokyo, Japan). X‐ray diffractometry (XRD) was performed with a D8 DISCOVER multi‐purpose X‐ray diffractometer (Bruker Daltonik GmbH, Bremen, Germany) at a 2*θ* range of 5°–80° and a scanning rate of 2° min^−1^. X‐ray photon spectroscopy (XPS) was performed using an Escalab 250Xi X‐ray electron spectrometer (Thermo Fisher Scientific, Waltham, MA, USA). Fourier transform infrared (FTIR) spectra in the region of 400–4000 cm^−1^ were recorded with an FT‐IR spectrometer (PerkinElmer, Inc., Waltham, MA, USA) or a VERTEX 70v FT‐IR spectrometer (Bruker Daltonik GmbH). The EPR analysis was performed to determine residual free radicals in deionized water using DMPO as a radical trapping agent. During the reaction process, 1mL sample was taken at 10 s, 1, 3, 5, 10, and 30min respectively and mixed with 1.0 mL of 8.8 mm DMPO solution. This solution was then transferred to a capillary tube for the detection of free radicals in EPR analysis.

### Animals

The animal study protocol was approved by the Animal Ethics Committee of Shanghai Tenth People's Hospital (Shanghai, China). All animal experiments were conducted with specific pathogen‐free *C57BL/6* mice aged 6–8 weeks.

### Cell Culture

Human primary liver cancer (PLC) cells were cultured in high glucose Dulbecco′s Modified Eagle′s Medium supplemented with 10% fetal bovine serum, 100 µg mL^−1^ of streptomycin, and 100 U mL^−1^ of penicillin, and incubated at 37 °C under an atmosphere of 5% CO_2_/95% air. The cells were passaged every 2–3 days and those in the logarithmic growth phase were used for experimentation.

### Flow Cytometry

The cells were lysed and collected together with the supernatant into a 10‐mL flow centrifuge tube. The number of cells per sample was set at 1–5 × 10^6^. After washing twice with autoclaved phosphate‐buffered saline (PBS), the cells were centrifuged at 1000 rpm for 5 min. Once the supernatant was discarded, the cells were resuspended in buffer solution containing 2 µL of Annexin V‐fluorescein isothiocyanate (FITC) and incubated at room temperature in the dark for 20 min. Afterward, 2 µL of propidium iodide (PI) were added and the mixture was incubated for 5 min in the dark prior to flow cytometry at excitation light wavelengths of 488 and 515 nm to detect FITC fluorescence, and 560 nm to detect PI. The data were analyzed using Flowjo7.6 software (Becton, Dickinson and Company, Franklin Lakes, NJ, USA).

### Cell Proliferation and Viability Assay

PLC cells were cultured in the wells of a 6‐well plate at a density of 1 × 10^5^/well. The experimental group was treated with 1000 µg mL^−1^ of ZnO_0.2_–NiO@COOH, while the control group was treated with the same volume of PBS for 24 h. Calcein AM fluorescent dye (2 µm) and PI reagent were warmed to room temperature for 30 min prior to use. Then, 5 µL of PI (16 mm) and 10 mL of PBS were mixed well to prepare an 8 µm PI solution. A working solution composed of 5 µL of calcein AM (4 mm) and 10 mL of PI was directly used for cell staining.

### Cell Proliferation Analysis

PLC cells in the logarithmic growth phase were added to the wells of a 96‐well plate at a concentration of 1 × 10^5^/mL. After the cells had adhered to the walls, the culture medium was discarded and the adhered cells were treated with different concentrations of ZnO_0.2_–NiO@COOH in triplicate wells for 0, 24, 48, 72, and 96 h. Then, 10 µL of *CCK‐8* reagent were directly added to each well and the plates were incubated for an additional 2 h. If the test substance had redox properties, the medium was replaced before adding the *CCK‐8* reagent. The optical density of the wells at a wavelength of 450 nm was determined with a multifunctional microplate reader.

### Living Animal Imaging Technology

ICG is a fluorescent dye with excitation and emission wavelengths around 785 and 810 nm, respectively. Combining ICG with ZnO‐NiO@COOH, the distribution and enrichment of the material in mice can be observed. ZnO‐NiO@COOH (2 mg) were stirred in deionized water solution of ICG (1 mg mL^−1^, 10 mL) overnight, and then divided from the mixture through centrifugation (12 000 rpm, 5 min), obtaining ZnO‐NiO@COOH‐I. In vivo imaging pictures were taken after ZnO‐Nio@COOH‐I was injected into mice via tail vein. In the experiment, the time points of 0, 0.5, 1, 2, 4, 12, and 24 h were selected for photographing and sampling (*n* = 3/group). The heart, spleen, lung, kidney, liver, stomach, and colorectum of the animals were taken out and photographed (*n =* 3/group). The injection dose of ZnO‐NiO@COOH was 100 mg kg^−1^. The animals were anesthetized with isoflurane.

### Hemolysis Analysis

PBS (0.5 mL) and fresh mouse blood (10 µL) were added to each of the 14 eppendorf tubes. Then, 0.5 mL of water was added to the first tube as a positive control and 0.5 mL of PBS was added to the second tube as a negative control, while 0.5 mL of ZnO_0.2_–NiO@COOH diluted with PBS to different concentrations was added to tubes 3–14. After shaking, the tubes were incubated at 37 °C for 1 h. A positive hemolysis test was determined by the addition of water to the first test tube causing all of the red blood cells to rupture, which indicated that the sample was not suitable for injection. The second tube was used to check the adhesion reaction and was negative for the hemolysis test.

### ROS Detection

Standards (50 µL) were added to the wells of microtiter plates. Then, 10 µL of the test sample and 40 µL of diluted sample were added to individual wells and the plate was incubated at 37 °C for 30 min. After washing five times, 50 µL of enzyme‐labeled reagent were added to each well and the plate was incubated at 37 °C for an additional 30 min. Then, after washing five times, 50 µL of solution A and 50 µL of solution B were added to the wells and the plate was incubated at 37 °C in the dark for 15 min for color development. Finally, 50 µL of stop solution was added to each well to stop the reaction. The optical density of each well was detected at a wavelength of 450 nm using a microplate reader. ROS ((DCFH‐DA) Assay Kit (Cat#S0033S, Beyotime, shanghai, china). Hydrogen peroxide solution (Hengjian Biological Company, Guangdong, China).

### Hematoxylin and Eosin (HE) Staining

See Supporting Information Materials and Methods 1 and 2 for details.

### Terminal Deoxynucleotidyl Transferase *dUTP* Nick‐End Labeling (TUNEL) Assay

See Supporting Information Materials and Methods 3 for details.

### The *mRNA* Analysis

See Supporting Information Materials and Methods 4–6 for details.

### Immunofluorescence

Frozen liver tissue sections were thawed at room temperature for 30 min, washed three times with PBS, blocked with 1*%* bovine serum albumin for 30 min, and washed three additional times with PBS. Afterward, the sections were incubated with primary antibodies (dilution, 1:100–200) for 1 h, then washed three times with PBS, incubated with secondary antibodies (dilution, 1:100) for 1 h, and washed three times with PBS. Following incubation with 4′,6‐diamidino‐2‐phenylindole (dilution, 1:1000) for 1 min, the sections were washed three times with PBS, sealed with glycerin, and observed under a fluorescence microscope.

### Enzyme‐Linked Immunosorbent Assay for Detection of Inflammatory Factors

The samples (25 µL) and diluted samples (75 µL) were added to the wells of a microtiter plate, which was incubated at room temperature for 2.5 h. After washing four times, 100 µL of biotinylated antibody was added to each well and the plate was incubated for an additional 1 h at room temperature. Afterward, 100 µL of streptavidin solution were added to each well and the plate was incubated at room temperature for 45 min. Then, after washing four times, 100 µL of TMB one‐step substrate reagent were added to each well and the plate was incubated for 30 min in the dark at room temperature. Finally, 50 µL of stop solution were added to each well and the optical density was measured at a wavelength of 450 nm using a microplate reader.

### Immunohistochemical Analysis

See Supporting Information Materials and Method 7 for details.

### Statistical Analysis

All experimental data were expressed as the mean ± standard deviation. Statistical analyses were conducted using GraphPad Prism 7 software (GraphPad Software, Inc., San Diego, CA, USA). The mean values of two groups were compared using the two‐tailed unpaired t‐test, while those of three or more groups were compared using analysis of variance. A probability (*p*) value of ≤ 0.05 was considered statistically significant.

## Conflict of Interest

The authors declare no conflict of interest.

## Supporting information

Supporting Information

## Data Availability

The data that support the findings of this study are available in the supplementary material of this article.
