# Supplementary material for: Scavenging ROS to Alleviate Acute Liver Injury by ZnO‐NiO@COOH
Source: Adv Sci (Weinh). 2022 Feb 9;9(11):2103982. doi: 10.1002/advs.202103982 (PMC9008431; doi:10.1002/advs.202103982)
Supplement: Supplementary file 1 — Supporting Information [file ADVS-9-2103982-s001.pdf]

ADVANCED SCIENCE
Open Access

Supporting Information

for *Adv. Sci.*, DOI 10.1002/advs.202103982

Scavenging ROS to Alleviate Acute Liver Injury by ZnO-NiO@COOH

Xuan Wu, Shiyu Liu, Huanhuan Zhu, Zili Ma, Xiaohu Dai and Weiwei Liu**

ADVANCED SCIENCE
Open Access

Supporting Information

for *Adv. Sci.,* DOI: 10.1002/advs.202103982

Scavenging *ROS* to alleviate acute liver injury by *ZnO*-*NiO@COOH*

XuanWu, Shiyu Liu, Huanhuan Zhu, Zili Ma, Xiaohu Dai, Weiwei Liu**

Supporting Information

Scavenging *ROS* to alleviate acute liver injury by *ZnO-NiO@COOH*

Xuan Wu[a,1], Shiyu Liu[b,c,1], Huanhuan Zhu[a], Zili Ma[a], Xiaohu Dai[b,c,*], Weiwei Liu[d,a*]

[a]Central Laboratory and Department of Laboratory Medicine, Shanghai Tenth People's Hospital, Tongji University, Shanghai, 200070, China

[b]State Key Laboratory of Pollution Control and Resource Reuse, School of Environmental Science and Engineering, Tongji University, Shanghai, 200092, China

[c]Shanghai Institute of Pollution Control and Ecological Security, Shanghai, 200092, China

[d]Department of Laboratory Medicine, Longhua Hospital, Shanghai University of Traditional Chinese Medicine, Shanghai, 200032, China.

*Correspondence

Xiaohu Dai, State Key Laboratory of Pollution Control and Resource Reuse, School of Environmental Science and Engineering, Tongji University, Shanghai, 200092, China. E-mail: daixiaohu@tongji.edu.cn.

Weiwei Liu, Department of Laboratory Medicine, Longhua Hospital, Shanghai University of Traditional Chinese Medicine, Shanghai, 200032, China. E-mail: huashanvivian@126.com.

"[1]" These authors contributed equally to this work.

Supplemental Materials and Methods 1

Embedding of tissue with paraffin

Fresh tissue samples were placed in 10% formalin for 24 *h* and dehydrated with a graded series of alcohol and xylene (75% alcohol, 2 *h* → 85% alcohol, 1 *h* → 95% alcohol I, 30 *min* → 95% alcohol II, 30 *min* → 100% alcohol I, 10 *min* → 100% alcohol II, 10 *min* → xylene I, 30 *min* → xylene Ⅱ, 30 *min* → paraffin Ⅰ, 2 *h* → paraffin wax Ⅱ, 2 *h*). Then, the embedded tissues were sliced to a thickness of 5 *μm* using a microtome.

Supplemental Materials and Methods 2

Hematoxylin & Eosin (*HE*) staining

Tissues slices were dewaxed by heating to 62°*C* in an oven for 2 *h* and then rehydrated in a graded series of xylene/alcohol/*PBS* (xylene I, 10 *min* → xylene II, 10 *min* → 100% alcohol Ⅰ, 5 *min* → 100% alcohol II, 5 *min* → 95% alcohol I, 5 *min* → 95% alcohol II, 5 *min* → 85% alcohol, 5 *min* → 75% alcohol, 5 *min* → *PBS* I, 5 *min*). Afterward, the tissues slices were stained with hematoxylin for 10 min. Color separation was performed by rinsing the tissue slices with 1% hydrochloric acid and alcohol (300 *mL* of 75% alcohol, 2 *mL* of concentrated *HCl*) for 30 *s* followed by running tap water for 5 *min*. Next, the tissues were stained with eosin dye solution for about 50 *s* and then rinsed with 95% alcohol. The slices were then subjected to a graded series of alcohol and xylene for dehydration and transparency (75% alcohol, 5 *min* → 85% alcohol, 5 *min* → 95% alcohol, 3 *min* → 100% alcohol, 3 *min* →

xylene I, 10 *min* → xylene II, 10 *min*). Afterward, the slices were mounted onto glass slides with neutral gum and observed under a microscope.

Supplemental Materials and Methods 3

TUNEL analysis

The tissue slices were dewaxed by heating to 62°*C* in an oven for 2 *h* and then rehydrated in a graded series of xylene/alcohol/*PBS* (xylene I, 10 *min* → xylene II, 10 *min*→ 100% alcohol I, 5 *min*→ 100% alcohol II, *min* → 95% alcohol I, 5 *min* → 95% alcohol II, 5 *min* → 85% alcohol, 5 *min* → 75% alcohol, 5 *min* → PBS I, 5 *min* → PBS II, 5 *min*). The slides were immersed in 3% H_2O_2 to block endogenous peroxidase activity, incubated for 20 *min* at room temperature, and then washed three times with *PBS* for 5 *min* each time. Following the addition of proteinase K at 37°*C* for 30 *min*, the slides were washed twice with *PBS* for 5 *min* each time.

The *TUNEL* reaction mixture consisted of 50 *μL* of *TdT* enzyme and 450 *μL* of *dUTP*.

Experimental group: 100 *μL* of the *TUNEL* reaction mixture.

Positive control group: 100 *μL* DNase I solution at room temperature for 10 *min*, then 100 *μL* of the *TUNEL* reaction mixture.

Negative control group: 50 *μL* of *dUTP* solution.

The sections were incubated at 37°*C* for *1* h in the dark. At the end of the reaction, the samples were washed three times with PBS for 5 *min* each time. Next, 50 *μL* of converter-POD working solution were added and the samples were incubated at 37°*C*

in the dark for 60 *min*, then washed with *PBS* three times for 5 *min* each time. Color was developed by dropwise addition of 50 *μL* of *DAB* color developing solution and reacted for 10 *min* at room temperature, then washed three times with *PBS* for 5 *min* each time. The nuclei were counterstained with hematoxylin. Finally, the samples were mounted on glass slides with neutral gum and observed under a microscope.

Supplemental Materials and Methods 4

The *mRNA* extraction

Before the experiment, all relevant instruments were treated with *DEPC* water to remove RNase and then autoclaved. The tissue fragments were placed in 1.5 *mL* Eppendorf tube and mixed with 1 *mL* of *Trizol* reagent, then placed on ice for 5 *min* to fully lyse the cells. Afterward, the tissues were disrupted by shaking with magnetic beads at 300 *rpm* for 5 *min*. Following centrifugation at 12,000 *rpm* at 4°*C* for 5 *min*, the supernatant was transferred to a new Eppendorf tube, then mixed with 200 *μL* of chloroform, shaken vigorously for 15 *s*, and placed on ice for 10 *min*. Afterward, the samples were centrifuged at 12,000 *rpm* and 4°*C* for 15 *min*. The homogenate in the tube was divided into three layers with the *RNA* dissolved in the colorless upper layer, which was transfer to a new eppendorf tube. Following the addition of an equal volume of isopropanol, the sample was mixed well and then incubated at room temperature for 10 *min*. Following centrifugation at 12,000 *rpm* at 4°*C* for 10 *min*, the supernatant was removed. Then, the *RNA* pellet was washed with 1 mL of 75% ethanol and centrifuged at 12,000 *rpm* and 4°*C* for 5 *min*. After the ethanol was

removed, the tube was incubated at 55°*C* for 10 *min*. Next, the *RNA* was suspended in 20–50 *μL* of RNase-free *DEPC* water. The concentration of the *RNA* was measured at 260 *nm* and purity at 280 *nm*, expressed as *A260/A280*. Finally, the samples were stored at -80°*C* until use.

Supplemental Materials and Methods 5

Reverse Transcription

RNA (2 *μg*), N6 primer (1 *μL*), and DEPC water to a final volume of 15 *μL* were mixed well in a 70°*C* water bath for 5 *min*, and then cooled on ice for 2 *min*. According to the measured *mRNA* concentration, the volume of *RNA* to be added was calculated. Each 10 *μL* reaction volume consisted of dNTPs (1 *μL*), 5 × buffer (5 *μL*), Rasin (1.25 *μL*), *DEPC* water (1.75 *μL*), and MLV (1 *μL*). The reaction was conducted in a 37°*C* water bath for 60 *min*.

Supplemental Materials and Methods 6

Real-time fluorescent quantitative *PCR*

The Gene IDs of the target genes of the required species were retrieved from the GeneBank database and the primers were obtained from PrimerBank (Supplemental Table 1). Each 10 *μL* reaction system consisted of *SYBR* Green I (dye (5 *μL*), forward primer (0.5 *μL)*, reverse primer (0.5 *μL*), *cDNA* (1 *μL*), and *DEPC* water (3 *μL*). After the above reaction system was fully mixed and centrifuged, the *PCR* reaction was carried out according to the following reaction conditions: pre-denaturation at 95°*C*

for 30 s followed by 40 cycles of amplification at 95°*C* for 5 s and 60°*C* for 32 *s*. Dissolution curve program: 95°*C* for 15 *s*, 60°*C* for 15 *s*, and 95°*C* for 15 *s* for 1 cycle.

The purity of the amplified products was determined by reference to the melting curve. According to dynamic analysis, the relative expression of target gene *mRNA* was calculated with the following formula: $2^{-\Delta\Delta Ct} = 2^{-\Delta Ct\ Object\ group - \Delta Ct\ control\ group}$

Supplemental Materials and Methods 7

Immunohistochemical analysis

The slices were dewaxed by heating to 60°*C* in an oven for 2 *h* and then deparaffinized with a graded series of xylene/ethanol/water (xylene, 10 *min* → xylene, 10 *min* → xylene, 10 *min* → absolute ethanol, 10 *min* → 95% ethanol, 5 *min* → 85% ethanol, 5 *min* → 75% ethanol, 5 *min* → ddH_2O, 5 *min*). Endogenous peroxidase was inactivated by immersing the slices in 3% $H_2 0_2$ for 30 *min*. Antigen retrieval was performed with acid retrieval solution (21.9 *g* of trisodium citrate, 3.6 *g* of citric acid, and ddH_2O to a final volume of 3,000 *mL*). Next, blocking was performed with 1% BSA at 37°*C* for 30 *min*. Then, the slices were incubated overnight with primary antibodies (dilution, 1:500–10,000) at 4°*C* followed by incubation with horseradish peroxidase-conjugated secondary antibodies at 37°*C* for 30 *min*. Color was developed with the use of DAB solution for 3–10 *min*. The nuclei were stained with hematoxylin for 10 min (differentiation 1–2 times). The slices were dehydrated with a graded series of ethanol/xylene/phenolic acid (75% ethanol, 5 *min* → 85% ethanol, 5 *min* →

95% ethanol, 10 *min* → absolute ethanol, 10 *min* → phenolic acid, 5 *min* → xylene, 10 *min* → xylene, 10 *min*). Afterward, the slices were sealed in resin and stored for later use.

Supplemental Table1

Gene		Primer sequence (5′-3′)
TNFα	Forward	AACTCCAGGCGGTGCCTATG
TNFα	Reverse	TCCAGCTGCTCCTCCACTTG
IL-1β	Forward	AGCTTCAGGCAGGCAGTATC
IL-1β	Reverse	TCATCTCGGAGCCTGTAGTG
IL-6	Forward	AAGTCCGGAGAGGAGACTTC
IL-6	Reverse	TGGATGGTCTTGGTCCTTAG
IL-10	Forward	CCTGGCTCAGCACTGCTATG
IL-10	Reverse	TCACCTGGCTGAAGGCAGTC
β-actin	Forward	CATGTACGTTGCTATCCAGGC
β-actin	Reverse	CTCCTTAATGTCACGCACGAT

Supplemental Table 2. Reagent details

	Reagent name	CAS	company
1	eBioscience^{TM}Amexin V-FITC Apop Kit 300 Tests	LOT 215819000	InvitrogenZ
2	FastStart Universal SYBR® Green	LOT 50837000	Roche

	Master(ROX)		
3	Random Primer d(N)e		BOGUANG
4	4*dNTP Mixture		BOGUANG
5	M-MLV Reverse Transcriptase	0000321392	PROMEGA
6	M-MLV RT 5*Buffer	0000318756	PROMEGA
7	Recombinant RNsasin® Ribonuclease Inhibitor	0000329876	PROMEGA
8	D- (+)-Galactosamive hydrochloride	G0500-5G	SIGMA
9	Lipopolysaccharides from Escherichia colio o111:B4	L4130	SIGMA
10	RayBio® Mouse IL-6 ELISA Kit		RayBio
11	RayBio® Mouse TNF-alpha ELISA Kit		RayBio
12	Reactive Oxygen Species Assay Kit	50101ES01	BIOLAI
13	Goat Anti-Rat IgG(H+L),HRP	ABS20031	absin
14	Caspase 3	19677-1-AP	PROTEINTECH
15	Caspase 9	9505S	CST
16	CD11b	ab133357	abcam
17	F4/80	ab16911	abcam

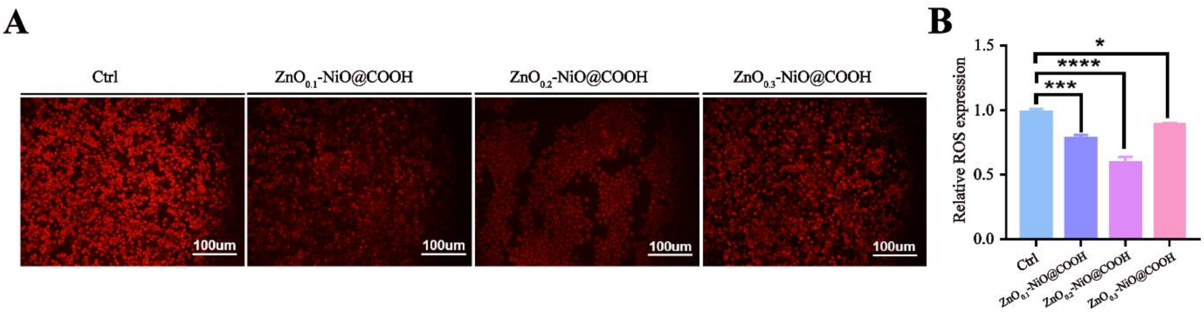

Supplyment Figure 1. Verification of particles uptake of *ROS*. A, Fluorescent probe to detect *ROS* in cells (*n=2*). B, Statistics of *ROS* fluorescence intensity (*n=3*, $^{*}p < 0.05$, $^{***}p < 0.001$, $^{****}p < 0.0001$).

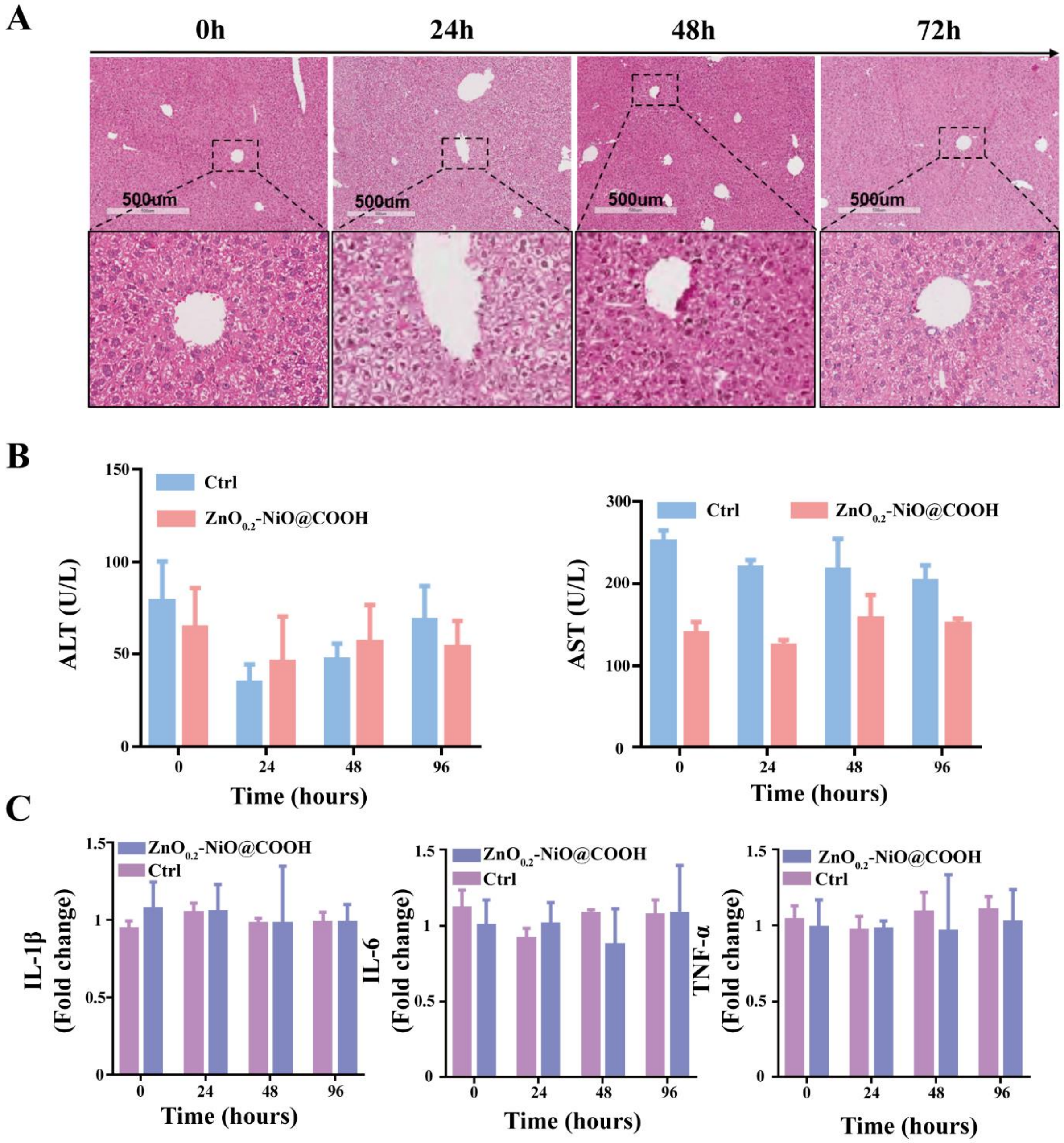

Supplyment Figure 2. Animal safety verification of *ZnO*$_{0.2}$*–NiO@COOH*. A, H*E staining* to observe liver tissue damage at 0, 24, 48, and 72 *h* (*n=5*). B, Detection of *ALT* and *AST* to assess liver function (*n=5*, $p > 0.05$). C, Liver tissue expression levels of inflammatory factors (*n=3*, $p > 0.05$).

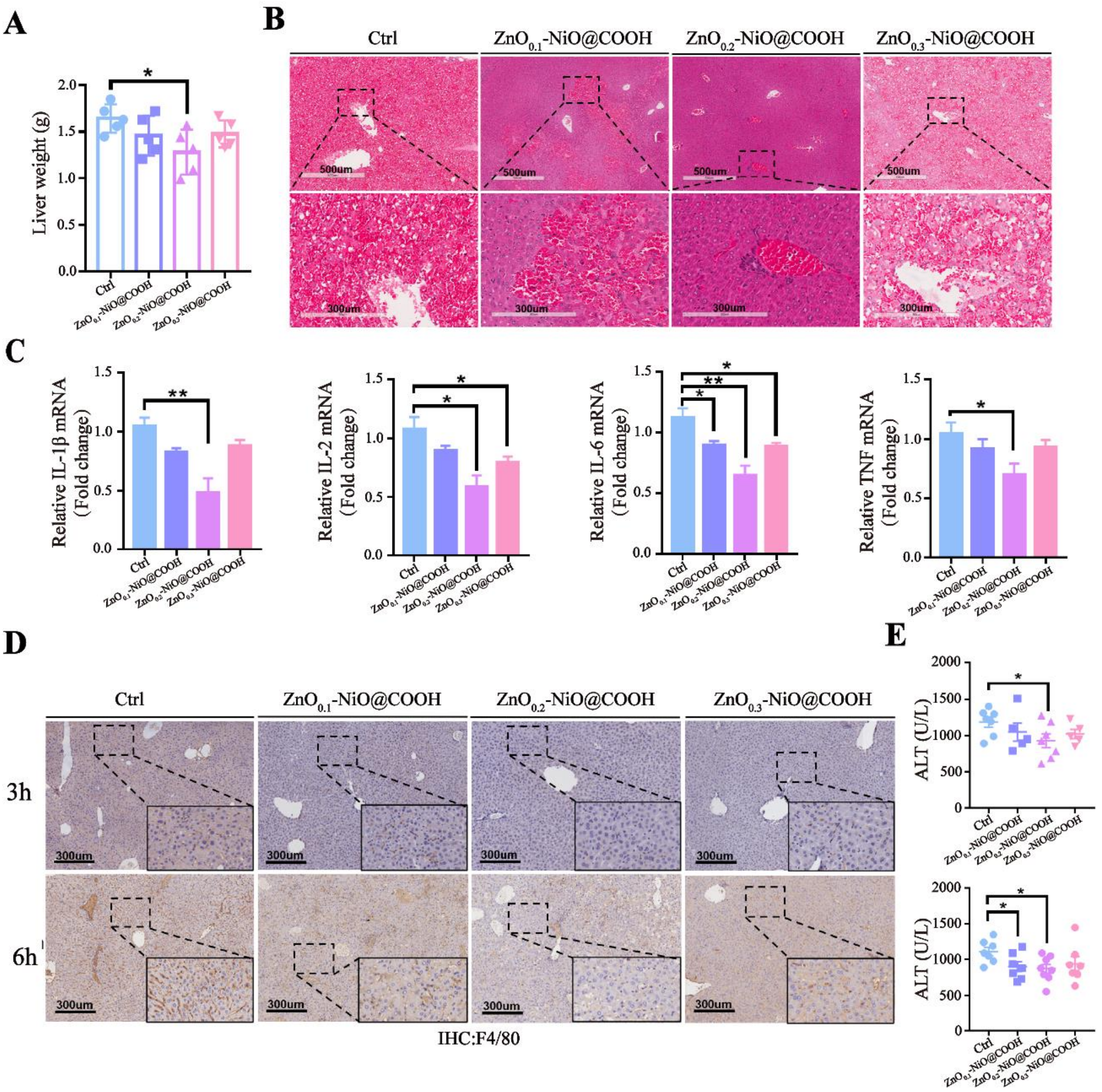

Supplyment Figure 3A-E. Verify the effect of particles on acute liver injury. A, Construct an *ALI* model and weigh the livers of four groups of mice after death (*n=5*, $^{*}p < 0.05$). B, *HE staining* of liver tissue. C and D, Detection of inflammatory factors in liver tissue by *PCR* and immunohistochemical staining (*n*=3, $^{*}p < 0.05$, $^{**}p < 0.01$). E, Detection of *ALT* and *AST* (*n=5-8*, $^{*}p < 0.05$).
